# Protein-Protein Interactions in Papillary and Nonpapillary Urothelial Carcinoma Architectures: Comparative Study

**DOI:** 10.2196/76736

**Published:** 2025-11-27

**Authors:** Charissa Chou, Yiğit Baykara, Sean Hacking, Ali Amin, Liang Cheng, Alper Uzun, Ece Dilber Gamsiz Uzun

**Affiliations:** 1 Department of Pathology and Laboratory Medicine, Rhode Island Hospital, 593 Eddy Street, Providence, RI, United States; 2 Department of Pathology and Laboratory Medicine, Alpert Medical School, Brown University, Providence, RI, United States; 3 Department of Pathology, University of Arizona, Tucson, AZ, United States; 4 Department of Pathology, Grossman School of Medicine, New York University, New York, NY, United States; 5 Brown Center for Clinical Cancer Informatics and Data Science, Providence, RI, United States; 6 Legorreta Cancer Center, Brown University, Providence, RI, United States; 7 Department of Pediatrics, Brown University, Providence, RI, United States; 8 Brown Center for Clinical Cancer Informatics and Data Science (CCIDS), Providence, RI, United States; 9 Center for Computational Molecular Biology (CCMB), Brown University, Providence, RI, United States

**Keywords:** urothelial carcinoma, comprehensive genomic profiling, protein-protein interactions, network biology, drug repurposing

## Abstract

**Background:**

Bladder cancer is a disease characterized by complex perturbations in gene networks and is heterogeneous in terms of histology, mutations, and prognosis. Advances in high-throughput sequencing technologies, genome-wide association studies, and bioinformatics methods have revealed greater insights into the pathogenesis of complex diseases. Network biology–based approaches have been used to identify complex protein-protein interactions (PPIs) that can lead to potential drug targets. There is a need to better understand PPIs specific to urothelial carcinoma.

**Objective:**

This study aimed to elucidate PPIs specific to papillary and nonpapillary urothelial carcinoma and identify the most connected or “hub” proteins, as these are potential drug targets.

**Methods:**

A novel PPI analysis tool, Proteinarium, was used to analyze RNA sequencing data from 132 patients with papillary and 270 patients with nonpapillary urothelial carcinoma from the TCGA Cell 2017 dataset and 39 patients with papillary and 88 patients with nonpapillary urothelial carcinoma from the TCGA Nature 2014 dataset. Hub proteins were identified in distinct PPI networks specific to papillary and nonpapillary urothelial carcinoma. Statistical significance of clusters was assessed using the Fisher exact test (*P*<.001), and network separation was quantified using the interactome-based separation score.

**Results:**

RPS27A, UBA52, and VAMP8 were the most connected or “hub” proteins identified in the network specific to the papillary urothelial carcinoma. In the network specific to the nonpapillary carcinoma, GNB1, RHOA, UBC, and FPR2 were found to be the hub proteins. Notably, GNB1 and FPR2 were among the proteins that have existing drugs targeting them.

**Conclusions:**

We identified distinct PPI networks and the hub proteins specific to papillary and nonpapillary urothelial carcinomas. However, these findings are limited by the use of transcriptomic data and require experimental validation to confirm the functional relevance of the identified targets.

## Introduction

According to the National Cancer Institute Surveillance, Epidemiology, and End Results program report, the estimated number of deaths from bladder cancer in the United States in 2025 was 17,420. Urothelial carcinoma contributes to more than 90% of bladder cancers and is more prevalent in men than in women [1]. Urothelial carcinomas can be divided into papillary and nonpapillary based on their architecture. Papillary urothelial carcinomas grow in slender projections with fibrovascular cores. Although most do not penetrate the deeper layers of the bladder (noninvasive papillary carcinoma), others can become invasive. Nonpapillary urothelial carcinomas do not grow toward the hollow center of the bladder and can be invasive or noninvasive (flat carcinoma in situ) [2]. Invasive nonpapillary urothelial carcinomas can aggressively infiltrate the bladder wall, leading to metastasis in lymph nodes and invade adjacent surrounding anatomical structures, such as the prostatic stroma, seminal vesicles, uterus, vagina, pelvic wall, and the abdominal wall. Previous studies have used gene expression profiling and whole-genome comparative genomic hybridization to provide evidence that papillary and nonpapillary urothelial carcinomas have distinct molecular origins and characteristics [3].

Network biology is an emerging paradigm that aims to understand the complex interactions between molecules at the cellular level [4]. Experimental studies describing biological networks demonstrate that they are not random and are characterized by well-known organizing principles. With the advancement of high-throughput screening methods, protein-protein interaction (PPI) networks have been discovered [5]. In addition, large-scale PPI maps showed that proteins involved in specific phenotypes often interact physically. Moreover, it was also shown that similar phenotypes share neighboring network environments not only in model organisms but also in humans [6-9]. Network biology can be used to identify molecular biomarkers for cancer identification and progression. At the proteomic level, PPI network analysis is used for the discovery of novel biomarkers and disease-related functional modules. Several studies based on transcriptomic or microarray data for bladder cancer have identified PPI networks based on differentially expressed genes or coexpressed genes in clusters [10-13]. Most of the time, differentially expressed genes between patients and healthy participants or among patients with different tumor stages are used as an input for building PPI networks. Despite the advances in PPI analysis algorithms, none of the current network analysis tools are designed to identify clusters of patients based on their PPI networks by using the data from individual patient samples. In order to address this, we developed a novel PPI analysis and visualization tool, Proteinarium [14]. Proteinarium is a multisample PPI tool that identifies clusters of samples with shared networks [14]. The Proteinarium tool builds PPI networks from genomic data for individual samples, measures network similarity between samples using the Jaccard distance, clusters them based on these similarities, and identifies shared PPI networks associated with a disease phenotype. In this study, we aimed to identify distinct PPI networks as well as hub proteins specific to papillary and nonpapillary urothelial carcinomas using the Proteinarium tool, leading to potential drug targets for those 2 architectures.

## Methods

### Datasets

We studied 2 datasets, including clinical and genomic data from patients with urothelial carcinoma. The datasets including The Cancer Genome Atlas (TCGA) Cell 2017 [15] and TCGA Nature 2014 [16] were obtained from the cBioPortal for Cancer Genomics [17,18]. Only patients with mRNA *z*-score data were included in the analysis. Patient groups were identified based on urothelial carcinoma architecture (papillary or nonpapillary), sex, patient status (alive, recurrent, or deceased), and cancer stage (stage I, II, III, and IV) using custom-made R scripts (R version 4.0.4). The TCGA Cell 2017 dataset [15] included 133 patients with papillary urothelial carcinoma and 273 patients with nonpapillary urothelial carcinoma. Of these patients, 132 with papillary and 270 with nonpapillary carcinomas had RNA-seq data and were included in the PPI network analysis. In the TCGA Nature 2014 dataset [16], there were 39 patients with papillary urothelial carcinoma and 88 patients with nonpapillary urothelial carcinoma with RNA-seq data. Histologic variants of urothelial carcinoma were not included in either dataset. For both studies, the data were downloaded as gene expression *z* scores.

In the phenotypic data analysis, sex, age, follow-up time, overall survival, disease-free time, pathological distant metastasis stage, survival status, disease-free status, histologic grade, pathological primary tumor stage, pathological regional lymph node stage, and stage grouping data were included in the analysis of the TCGA Cell 2017 dataset. For the TCGA Nature 2014 dataset, patient data such as sex, age, survival status, overall survival, disease-free status, and disease-free time were included. A total of 346 and 129 patients with urothelial carcinoma were analyzed in the TCGA Cell 2017 and TCGA Nature 2014 datasets, respectively. Patients without phenotypic data were excluded from the analysis. Thus, there were differences in the number of patients in the PPI network analysis and phenotypic data analysis.

### Proteinarium Analysis and Network Construction

We used the Proteinarium tool to identify shared PPI networks in patients with papillary and nonpapillary urothelial carcinoma using the RNA-seq data from the TCGA Cell 2017 and TCGA Nature 2014 datasets. For each patient, we ranked the genes based on their *z* scores to identify the upregulated genes. To determine the number of seed genes used to build the PPI networks of each patient, we applied cophenetic correlation scores to the dendrogram output from the Proteinarium tool comparison of patients with papillary and nonpapillary urothelial carcinomas using the top 50, 100, 125, 150, 200, 250, and 300 seed genes. For papillary versus nonpapillary comparisons in both the TCGA Cell 2017 and TCGA Nature 2014 datasets, the cophenetic correlation coefficients remained stable after increasing the number of seed genes to 125, indicating that the overall dendrogram structure was not substantially altered by the larger number of seed genes (Figures S1-S3 in Multimedia Appendix 1). Therefore, we used the top 125 most upregulated seed genes for each patient as inputs for the Proteinarium tool. We performed the same cophenetic correlation score analysis for the downregulated genes in the TCGA Cell 2017 dataset (Figure S4 in Multimedia Appendix 1). Downregulated genes were chosen by ranking the genes from lowest to highest *z* score, then choosing the top N number of seed genes.

As a first step, the Proteinarium tool converts gene names to their protein names and uses PPI information from the STRING Database [19]. It builds the network graphs based on the seed genes for each patient. It calculates the similarity between each pair of graphs by using the Jaccard distance and records it in a matrix. Proteinarium uses this similarity matrix as an input for clustering graphs. The unweighted pair group method with arithmetic mean was used for clustering. Patients were clustered based on their network similarities as an output. The significance of clusters was assessed using the Fisher exact test, based on the relative abundance of cases and controls within each cluster. Networks of the significant clusters can be visualized and analyzed. In the Proteinarium analysis, we used the default parameters of the tool for building the graphs, and the maximum path length parameter was set to 2. By applying this, we included only the seed proteins connected directly to each other or via a single intermediary protein referred to as an imputed protein.

The analysis was performed using the following configuration settings: maximum number of vertices to render: 50, meta cluster threshold: 0.8, number of bootstrapping rounds: 0, maximum path length: 2, and repulsion constant: 1.2. Proteinarium was executed using the following command: java -jar <path to Proteinarium.jar> <arguments>, where <arguments> includes user-defined input files and options consistent with the parameters described earlier. A reproducibility package is provided in Multimedia Appendix 2, which includes (1) per-patient seed gene lists, (2) the exact Proteinarium command and configuration file, (3) the final network edge lists, and (4) the README file. The Proteinarium repository is available on GitHub [14].

The primary aim of the Proteinarium analysis was to identify hub and unique proteins in the PPI networks specific to papillary and nonpapillary urothelial carcinoma. Hub proteins were further examined using a drug repurposing tool to identify potential therapeutics, as targeting these highly connected nodes may disrupt critical interactions within the network. Unique proteins were evaluated through enrichment analysis to determine biological pathways significantly associated with each network.

### Network Annotation and Modularity

Networks of the significant clusters were annotated by g:Profiler, a web server for identifying enriched biological categories [20]. The unique proteins specific to the papillary and nonpapillary PPI networks were analyzed using g:Profiler to identify enriched gene ontology terms and the Kyoto Encyclopedia of Genes and Genomes (KEGG) pathways. Only those with a false discovery rate less than 0.05 were included. GEPHI version 0.9.2, a network visualization tool, was used to visualize the networks and their network-specific modularity [21]. We used the modularity function that was based on the Blondel algorithm on GEPHI [22]. Modularity is defined as a measure of network structure that identifies the strength of division of a network into modules.

### Separation Test

Separation testing is a computational method to determine the genetic similarity between 2 groups by comparing their PPI networks in the interactome. We performed separation tests to determine the distance between the networks specific to patients with papillary and nonpapillary urothelial carcinomas in the interactome using a Python script adapted from Menche et al [23]. The interactome is composed of 141,296 experimentally determined physical interactions between 13,460 proteins. s_AB_ is a measure of separation between the networks of 2 groups in the interactome. s_AB_>0 indicates a topological separation of the networks specific to 2 groups, and those groups can be regarded as distinct molecular entities. s_AB_<0 indicates that the networks specific to 2 groups overlap in the interactome.

### Connectivity Map and Drug Repurposing Tool

We used the connectivity map (CMap) from the Broad Institute and applied the repurposing tool to explore potential drugs that may target the hub genes of networks A, B, and C for CMapap [24]. CMap-L1000 Build 03 was queried with the genes of interest from networks A, B, and C; compounds with |τ| >90 were considered hits.

### Statistical Analysis

The chi-square or Fisher exact test were used for categorical variables when appropriate. Paired *t* tests (2-sided) were used to compare the means of pairs of groups. All statistical analyses were carried out using JMP Pro (version 16.0; SAS Institute). *P* values <.05 were considered to be statistically significant.

### Ethical Considerations

In this study, we used publicly available, deidentified datasets obtained from cBioPortal. Because all data were fully anonymized and accessible to the public, institutional review board approval was not required.

## Results

### Clinicopathologic Characteristics of the Study Cohorts

Of all the 346 patients with urothelial carcinoma in the TCGA Cell 2017 dataset [15], no significant differences were found between patients with papillary and nonpapillary urothelial carcinoma regarding sex, age, follow-up time, overall survival, disease-free time, and pathological distant metastasis stage. However, statistically significant differences were observed in survival status, disease-free status, histologic grade, pathological primary tumor stage, pathological regional lymph node stage, and stage grouping (Table 1). In 129 patients with urothelial carcinoma in the TCGA Nature 2014 dataset [16], no significant differences were found between the patients with papillary and nonpapillary carcinoma regarding age, overall survival, disease-free status, and disease-free time. However, statistically significant differences were observed in terms of sex and survival status (Table 2).

**Table 1.** Clinical parameters of urothelial carcinomas in the Cancer Genome Atlas Cell 2017 dataset.

Parameter	Patients with papillary carcinoma	Patients with nonpapillary carcinoma	*P* value
Sex, n/n (%)			.13
Female	27/133 (20.3%)	60/213 (28.2)	
Male	106/133 (79.7%)	153/213 (71.8)	
Age (y), mean (SEM)	66.9 (1.0)	68.4 (0.7)	.21
Follow-up time^a^ (d), mean (SEM)	521.8 (69.8)	652.5 (72.5)	.21
Survival status, n/n (%)			<.001
Living	90/133 (67.7)	104/213 (48.8)	
Deceased	43/133 (32.3)	109/213 (51.2)	
Overall survival^b^ (mo), mean (SEM)	24.4 (2.0)	27.1 (1.9)	.34
Disease-free status^c^, n/n (%)			<.001
Disease-free	75/108 (69.4)	79/167 (47.3)	
Recurred or progressed	33/108 (30.6)	88/167 (52.7)	
Disease-free time^d^ (mo), mean (SEM)	22.8 (2.2)	26.8 (2.3)	.23
Histologic grade^e^, n/n (%)			<.001
Low-grade	18/131 (13.7)	1/213 (0.5)	
High-grade	113/131(86.3)	212/213 (99.5)	
Pathological primary tumor stage, n/n (%)^f^			<.001
T1 or T2	56/118 (47.5)	53/199 (26.6)	
T3	41/118 (34.7)	117/199 (58.8)	
T4	21/118 (17.8)	29/199 (14.6)	
Pathological regional lymph node; stage, n/n (%)			.03
N0	89/116 (76.7)	117/187 (62.6)	
N1	8/116 (6.9)	27/187 (14.4)	
N2	19/116 (16.4)	43/187 (23.0)	
Pathological distant metastasis stage, n/n (%)			>.99
M0	75/79 (94.9)	89/93 (95.7)	
M1	4/79 (5.1)	4/93 (4.3)	
Stage grouping^g^, n/n (%)			<.001
Stage I^h^ or II	61/129 (47.2)	53/213 (24.9)	
Stage III	34/129 (26.4)	84/213 (39.4)	
Stage IV	34/129 (26.4)	76/213 (35.7)	

a Follow-up time was not available for 26 patients with papillary urothelial carcinoma and 68 patients with nonpapillary urothelial carcinoma; *t* test was performed for the available data in patients with papillary urothelial carcinoma (n=107) and patients with nonpapillary urothelial carcinoma (n=145).

b Overall survival was not available for 3 patients in patients with papillary urothelial carcinoma; a *t* test was performed for the available data in patients with papillary urothelial carcinoma (n=130) and nonpapillary group (n=213).

c Disease-free status was not available for 25 patients in the papillary and 46 patients in the nonpapillary group; a chi-square test was performed for the available data in patients with papillary urothelial carcinoma (n=108) and patients with nonpapillary urothelial carcinoma (n=167).

d Disease-free time was not available for 26 patients with papillary and 46 patients with nonpapillary carcinoma; a *t* test was performed for the available data in patients with papillary carcinoma (n=107) and patients with nonpapillary carcinoma (n=167).

e Histologic grade was not available for 2 patients with papillary carcinoma; a chi-square test was performed for the available data in patients with papillary urothelial carcinoma (n=131) and patients with nonpapillary carcinoma (n=213).

f There were 1 T1 stage patient, 15 Tx patients, 12 Nx patients, and 54 Mx patients among patients with papillary urothelial carcinoma and 1 T1 patient, 14 Tx patients, 24 Nx patients, and 120 Mx patients among patients with nonpapillary carcinoma.

g Stage information was unavailable for 2 patients in the papillary group; a chi-square analysis was conducted using the available data (papillary: n=131, nonpapillary: n=213).

h There was only 1 patient with a T1 stage tumor among patients with papillary urothelial carcinoma and 1 patient with T1 stage tumor among patients with nonpapillary urothelial carcinoma.

**Table 2.** Clinical parameters of urothelial carcinomas in the Cancer Genome Atlas Nature 2014 dataset.

Parameter	Patients with papillary urothelial carcinoma (n=41)	Patients with nonpapillary urothelial carcinoma (n=88)	*P* value
Sex, n (%)			.03
Female	5 (12.2)	27 (30.7)	
Male	36 (87.8)	61 (69.3)	
Age (y), mean (SEM)	67.5 (1.8)	69.0 (1.0)	.45
Survival status			.006
Living, n (%)	33 (80.5)	49 (55.7)	
Deceased, n (%)	8 (19.5)	39 (44.3)	
Overall survival (mo), mean (SEM)	14.6 (3.3)	19 (2.7)	.33
Disease-free status^a^, n (%)			.15
Disease-free	20 (62.5)	37 (46.2)	
Recurred or progressed	12 (37.5)	43 (53.8)	
Disease-free time^b^ (mo), mean (SEM)	9.0 (1.8)	11.0 (1.5)	.49

a Disease-free status was not available for 9 patients with papillary and 8 patients with nonpapillary carcinoma; chi-square test was performed for the available data in patients with papillary urothelial carcinoma (n=32) and nonpapillary group (n=80).

b Disease-free time was not available for 30 patients in papillary and 54 patients in nonpapillary group; *t* test was performed for the available data in patients with papillary urothelial carcinoma (n=11) and nonpapillary group (n=34).

### Proteinarium Identifies Distinct PPI Networks in Urothelial Carcinoma

We performed Proteinarium analysis using RNA-seq data from patients with papillary and nonpapillary urothelial carcinoma to compare their PPI networks. We analyzed the top 125 upregulated genes obtained from RNA-seq data from TCGA Cell 2017 [15] and TCGA Nature 2014 [16] datasets, as well as the top 125 downregulated genes from the TCGA Cell 2017 dataset.

**Figure 1.**
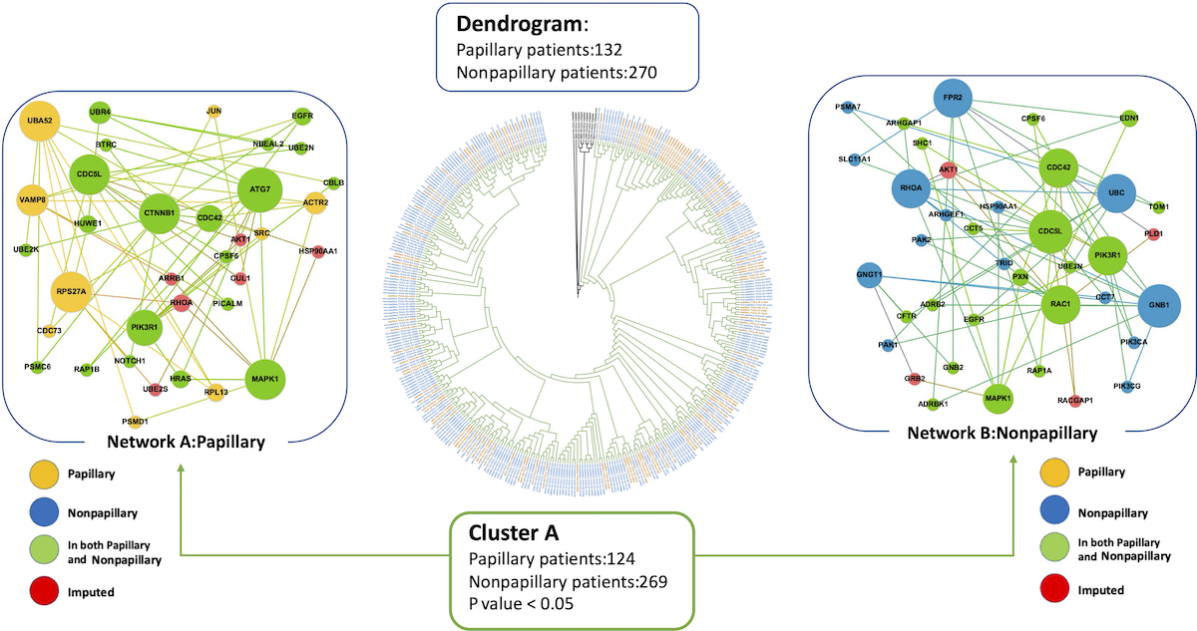
Dendrogram and consensus networks specific to papillary and nonpapillary urothelial carcinoma from The Cancer Genome Atlas Cell 2017 dataset (upregulated genes). The dendrogram shows 402 patients who were clustered based on their network similarities. Patients with green branches are part of the significant cluster whose networks are next to the dendrogram. In the networks, larger circles represent more connected nodes. Proteins specific to the papillary and nonpapillary groups are shown in yellow and blue, respectively. Proteins in both groups are shown in green. Proteins in red were imputed from the network analysis.

**Figure 2.**
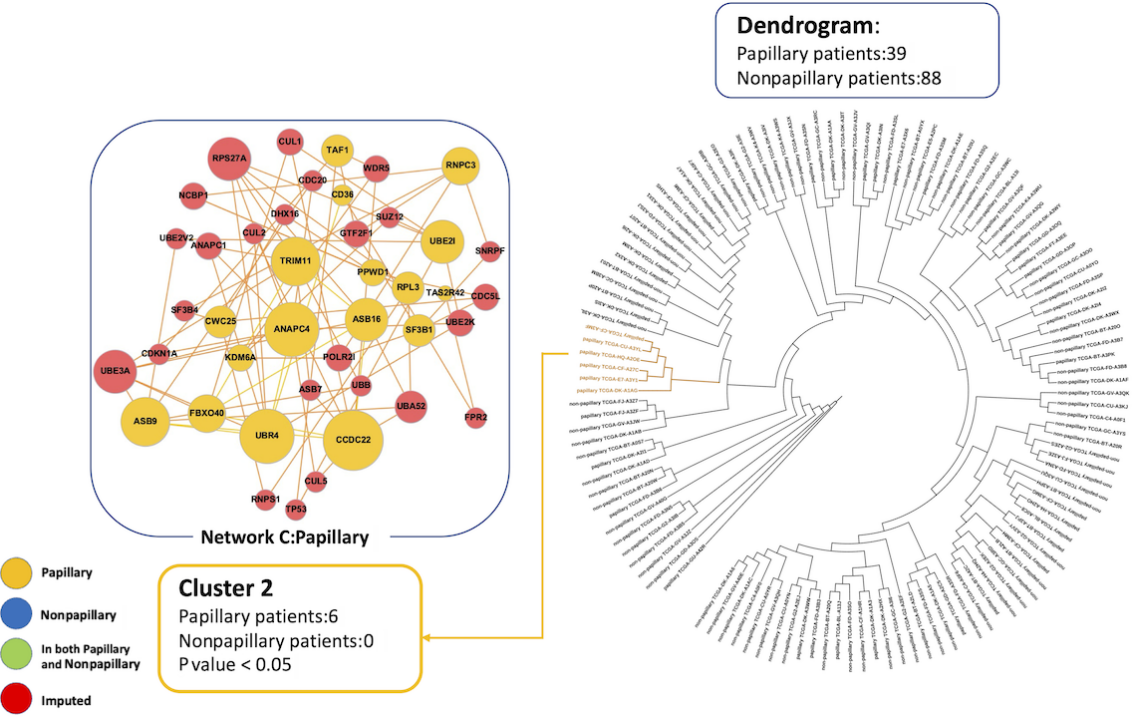
Dendrogram and consensus networks specific to papillary and nonpapillary urothelial carcinoma from The Cancer Genome Atlas Nature 2014 dataset (upregulated genes). The dendrogram shows 127 patients, who were clustered based on their network similarities. Patients with yellow branches are part of the significant cluster, whose network is shown to the left of the dendrogram. In the networks, larger circles represent more connected nodes. Proteins specific to the papillary and nonpapillary groups are shown in yellow and blue, respectively. Proteins shared by both groups are shown in green (none were identified in this dataset). Proteins in red were imputed from the network analysis.

In the analysis of the TCGA Cell 2017 dataset, 1 significant cluster (n=393; *P*<.001) was identified for further analysis, which included 89 (67.4%) of 132 patients with papillary urothelial carcinoma and 214 (79.3%) of 270 patients with nonpapillary urothelial carcinoma (Figure 1). In the network specific to papillary urothelial carcinoma (network A), RPS27A, UBA52, and VAMP8 were the most connected or hub proteins identified. In the network specific to nonpapillary carcinoma (network B), GNB1, RHOA, UBC, and FPR2 were found to be the hub proteins (Figure 1). In the analysis of the TCGA Nature 2014 dataset, 1 cluster of 6 patients with papillary urothelial carcinoma (network C) was identified as a significant cluster based on Fisher exact test (*P*<.001; Figure 2). In this PPI network, CCDC22, ANAPC4, and UBR4 were identified as hub proteins unique to papillary urothelial carcinoma. Six proteins were shared between the 2 papillary urothelial carcinoma consensus networks (networks A and C), including UBR4, CUL1, UBE2K, CDC5L, UBA52, and RPS27A.

We calculated the separation score between the papillary- and nonpapillary-specific networks from the TCGA Cell 2017 dataset (networks A and B). Using only the seed genes unique to each network, the separation score was s_AB_=0.1722. This value indicated that the papillary and nonpapillary consensus networks contained protein modules that did not overlap in the interactome and can therefore be considered distinct. Per Menche et al [23], strongly segregated phenotypes, such as mismatch repair-deficient and mismatch repair-intact breast cancer, have s_AB_ of approximately 0.32. Thus, an s_AB_ equal to 0.1722 reflects a moderate yet biologically meaningful network separation. The separation score comparing networks A and C was calculated as s_AB_=–0.0661. The negative s_AB_ value demonstrated that there was an overlap between the consensus PPI networks specific to papillary urothelial carcinoma using 2 independent datasets.

We analyzed PPI using the 125 most downregulated genes (Figure S4 in Multimedia Appendix 1). In the downregulated consensus networks, proteins unique to patients with papillary urothelial carcinoma included APP, RTP2, TP53, TAS2R13, SPTAN1, EPHB2, HIST1H2BH, MAPK9, and TAS2R42, whereas proteins unique to the PPI network specific to nonpapillary urothelial carcinoma included ANAPC13, LNX1, TAS2R46, REEP5, UBE2V2, UBB, EP300, and GPR31. Negative separation score (s_AB_=–1.4706) indicated an overlap between these 2 PPI networks.

Given the distinct genetic differences between low-grade and high-grade urothelial carcinomas, we compared the PPI networks of patients with high-grade papillary carcinoma (n=114) and high-grade nonpapillary carcinoma (n=267; Figure S5 in Multimedia Appendix 1). Several proteins were shared between high-grade papillary and nonpapillary consensus networks, suggesting shared genetic features of high-grade urothelial carcinomas. The hub proteins shared between the high-grade papillary and nonpapillary networks included UBA52, CDC5L, CDC42, PIK3R1, and MAPK1. The proteins unique to the high-grade papillary network included SRC, ACTR2, WASL, CDC73, PSMD1, and JUN. The proteins unique to the network specific to high-grade nonpapillary urothelial carcinoma included GNB1, UBC, FPR2, GNGT1, RHOA, PIK3CA, PIK3CG, PXN, UBE2N, SLC11A1, CCT7, HSP90AA1, and DOCK4. The separation score comparing the high-grade papillary and nonpapillary urothelial carcinoma-specific PPI networks was −0.2739, demonstrating the overlap between these networks.

We annotated network modularity for patients with papillary and nonpapillary urothelial carcinoma in both datasets by using GEPHI, a network and graph visualization tool. In this context, modules represented clusters of proteins that were more densely connected to each other than to the rest of the network. We found 5 modules in which proteins were grouped by common functionality for each network in networks A and C, and 6 modules in network B. We used the default setting in GEPHI. gProfiler was used to annotate the modules for networks A, B, and C to identify significant Gene Ontology (GO) terms and KEGG pathways [20]. Notably, the papillary networks from both datasets (networks A and C) contained 1 module enriched in proteins involved in ubiquitin-related activities. The KEGG pathway “Ubiquitin-mediated proteolysis” and the molecular function GO terms “ubiquitin-like protein conjugating enzyme activity” as well as “ubiquitin protein ligase binding” were common to the module found in networks A and C.

### Pathway and Gene Set Enrichment Analysis

We used the g:GOSt functional profiling tool from g:Profiler to visualize significant GO terms and KEGG pathways for the genes in networks A, B, and C [20] to visualize significant GO terms and KEGG pathways for the genes in networks A, B, and C (Table S1, S2, and S3 in Multimedia Appendix 3). The proteins unique to nonpapillary urothelial carcinoma (network B) were enriched in the PI3K-Akt signaling pathway (KEGG pathway, q=o.0040), with the most significant molecular function GO term being phosphatidylinositol-4,5-bisphosphate-3-kinase activity. The GO terms and KEGG pathways related to the PI3K-Akt signaling pathway were unique to network B. Ubiquitin-mediated proteolysis was the most significant KEGG pathway for network C (q=2.742e-12), and ubiquitin-like protein ligase binding was the most significant molecular function term for network A (q=0.00110187).

### Drug Repurposing

GNB1, the hub protein unique in the network specific to nonpapillary urothelial carcinoma, encodes the beta subunit of G proteins, which modulate transmembrane signaling, including that of the PI3K/AKT signaling pathway [25]. Using the CMap drug repurposing tool, we identified Y16 as a potential drug to target GNB1. Y16 is a rho-associated kinase inhibitor in preclinical trials. Other rho-associated kinase inhibitors, including Y-27632 and HA-1077, were shown to inhibit the proliferation and invasion of urothelial carcinoma cells and induce apoptosis, respectively [26,27]. FPR2, another hub protein unique in the networks specific to nonpapillary urothelial carcinoma, encodes the formyl peptide receptor 2 and is involved in the response to amyloid beta, regulation of the defense response, and positive regulation of monocyte chemotaxis [28]. TC-FPR2-43 is a formyl peptide receptor agonist in preclinical trials, which may be considered for use in treating nonpapillary urothelial carcinoma. We also identified drugs targeting other proteins unique to nonpapillary urothelial carcinoma, including PIK3CA and PAK1. PIK3CA*,* which encodes the p100α catalytic subunit of PI3K [29], is the target of several PI3K inhibitors used to treat breast cancer (alpelisib), follicular lymphoma (copanlisib), and chronic lymphocytic leukemia (idelalisib). PAK1, a member of the serine/threonine p2-activating kinase family, is targeted by several drugs in preclinical trials—serine/threonine kinase inhibitor FRAX486, p21 activated kinase inhibitors IPA-3 and NVS-PAK1-1, and rho-associated kinase inhibitor RKI-1447.

## Discussion

### Principal Findings 

The genomic landscape distinguishing papillary and nonpapillary urothelial carcinoma architectures is not completely understood. Using a network biology approach, we sought to understand how the PPI networks differed between patients with papillary and nonpapillary urothelial carcinoma. Proteinarium, a multisample PPI analysis tool, was used to analyze gene expression data from patients with papillary and nonpapillary urothelial carcinoma and identify the distinct PPI networks and hub proteins specific to these 2 architectures. Using the CMap drug repurposing tool, we identified known drugs that target the identified hub proteins. Our results showed that RPS27A, UBA52, and VAMP8 were the hub proteins in the network specific to papillary urothelial carcinoma (network A). This PPI network shared 6 proteins with network C, also specific to papillary urothelial carcinoma, obtained using the validation dataset. These 6 proteins—UBR4, CUL1, UBE2K, CDC5L, UBA52, and RPS27A—were enriched in gProfiler for ubiquitination processes, including ubiquitin-mediated proteolysis. Dysregulation of the ubiquitin-proteasome system, which regulates tumor suppressors and oncogenic proteins, is observed across cancer types [30]. In the PPI network specific to nonpapillary urothelial carcinoma, network B, we identified GNB1, RHOA, UBC, and FPR2 as hub proteins, which were involved in the PI3K/AKT signaling pathway. Additionally, we compared the PPI networks specific to high-grade papillary and nonpapillary urothelial carcinoma. Networks F and G (Figure S5 in Multimedia Appendix 1) exhibited an overlap. This finding is consistent with the shared genetic drivers that underlie high-grade tumors.

### Comparison to Prior Work

Of the proteins shared by networks A and C, the 2 PPI networks specific to papillary urothelial carcinoma, CDC5L and CUL1, have been found to be upregulated in urothelial carcinoma in previous studies. It has been shown that their overexpression is significantly associated with poorer prognosis in patients diagnosed with urothelial carcinoma [31,32]. Knockdown of CDC5L inhibited the proliferation of urothelial carcinoma cells by inducing apoptosis and limiting bladder cancer cell migration, invasion, and epithelial-mesenchymal transition [32]. Currently, there are no drugs in development that target CDC5L or CUL1. RPS27A and UBA52, which are hub proteins unique to papillary urothelial carcinoma in network A, have been found to be overexpressed in colon cancer [33], prostate cancer [34], and leukemia [35]. RPS27A and UBA52 are both ribosomal fusion proteins composed of ubiquitin conjugated to the ribosomal proteins S27a and L40, respectively. RPS27A, an RNA-binding protein part of the ribosomal 40S subunit [36], plays a role in inhibiting apoptosis, regulating the progression of the cell cycle, and promoting proliferation [35].

UBA52 was identified as a hub protein in the network specific to papillary urothelial carcinoma from the TCGA Cell 2017 dataset, and it also emerged as a shared hub protein in both high-grade papillary and nonpapillary urothelial carcinoma-specific PPI networks. UBA52 encodes a ubiquitin-ribosomal fusion protein involved in essential processes such as protein synthesis and degradation, both of which are upregulated in rapidly proliferating cancers. UBA52 is required for embryonic development and regulates protein synthesis and the cell cycle by modulating the expression of cyclins D1 and D3 [37]. Other shared hub proteins, including MAPK1, CDC42, and PIK3R1, are also involved in key signaling and regulatory pathways that support tumor progression.

PIK3CA is a protein in the nonpapillary urothelial carcinoma-specific PPI network [29]. PIK3CA upregulation has been implicated in bladder cancer by promoting cell growth and regulating metastasis [38-40]. Specifically, knockdown of PIK3CA was found to substantially inhibit cell proliferation [40], whereas overexpression of PIK3CA promoted bladder cancer cell growth, migration, invasion, and metastasis [39]. Higher levels of PIK3CA expression were associated with worse prognosis in bladder cancer [39]. Subsequent activation of PI3K at the plasma membrane by dimerized receptor tyrosine kinases causes phosphorylation of phosphatidylinositol 4,5-bisphosphate to produce the secondary messenger phosphatidylinositol 3,4,5-trisphosphate. Several PI3K inhibitors have been developed to treat breast cancer (alpelisib), follicular lymphoma (copanlisib), and chronic lymphocytic leukemia (idelalisib). PI3K inhibition has been found to suppress the growth, migration, and colony formation of bladder cancer cells in vitro [41].

RHOA is a unique hub protein in the PPI network specific to the nonpapillary urothelial carcinoma. Overexpression of RHOA, a member of the rho family of small guanosine triphosphatases that regulates cell attachment, motility, and shape [29], has been associated with bladder cancer cell proliferation and metastasis [41-43]. Ras-induced RhOA and NF-kappaB activation were implicated in promoting the invasion and migration of bladder cancer cells [44,45]. Three of the 4 hub proteins of network B, the PPI network specific to nonpapillary urothelial carcinoma—GNB1, RHOA, and FPR2—have been found to be associated with worse prognosis in gastric cancer [46], bladder cancer [42], lung cancer [47], and cervical squamous cell carcinoma [48].  

### Limitations

There are several limitations in this study. First, given tumor-node-metastasis (TNM) staging data were not available for the TCGA Nature 2014 dataset, and 344 (99.4%) out of 346 patients in the TCGA Cell 2017 dataset were muscle invasive (T2, T3, and T4). Therefore, these findings may not apply to nonmuscle invasive disease (T1) and should not be generalized. Second, the data were based on *z* scores of RNA-seq data. Although RNA transcript abundance reflects relative protein abundance, it is not a direct measurement of the quantity of each specific protein. By using *z* scores rather than absolute RNA transcript counts, we were able to draw conclusions about how a gene is upregulated or downregulated relative to the rest of the samples.

### Conclusions

This study identified 2 distinct PPI networks associated with the molecular architecture of papillary and nonpapillary urothelial carcinoma, suggesting that these architectures represent biologically distinct entities driven by different molecular mechanisms. The hub proteins within each network may serve as potential targets for papillary and nonpapillary urothelial carcinoma. Further studies are required to determine the efficacy of these identified drug targets.

## Supplementary material

10.2196/76736 Multimedia Appendix 1 Details on protein-protein interaction network analysis and cophenetic scores.

10.2196/76736 Multimedia Appendix 2 Input data for the reproducibility of protein-protein interaction network analysis.

10.2196/76736 Multimedia Appendix 3 Significant Gene Ontology terms and Kyoto Encyclopedia of Genes and Genomes pathways enriched in networks A, B, and C.

## Abbreviations

CMap: connectivity map
GO: Gene Ontology
KEGG: Kyoto Encyclopedia of Genes and Genomes
PPI: protein-protein interaction
RNA-seq: RNA sequencing
TCGA: The Cancer Genome Atlas
