# Supplementary material for: Protein-Protein Interactions in Papillary and Nonpapillary Urothelial Carcinoma Architectures: Comparative Study
Source: JMIR Bioinform Biotechnol. 2025 Nov 27;6:e76736. doi: 10.2196/76736 (PMC12661593; doi:10.2196/76736)
Supplement: Multimedia Appendix 1 [file bioinform-v6-e76736-s001.pdf]

Supplementary Figures

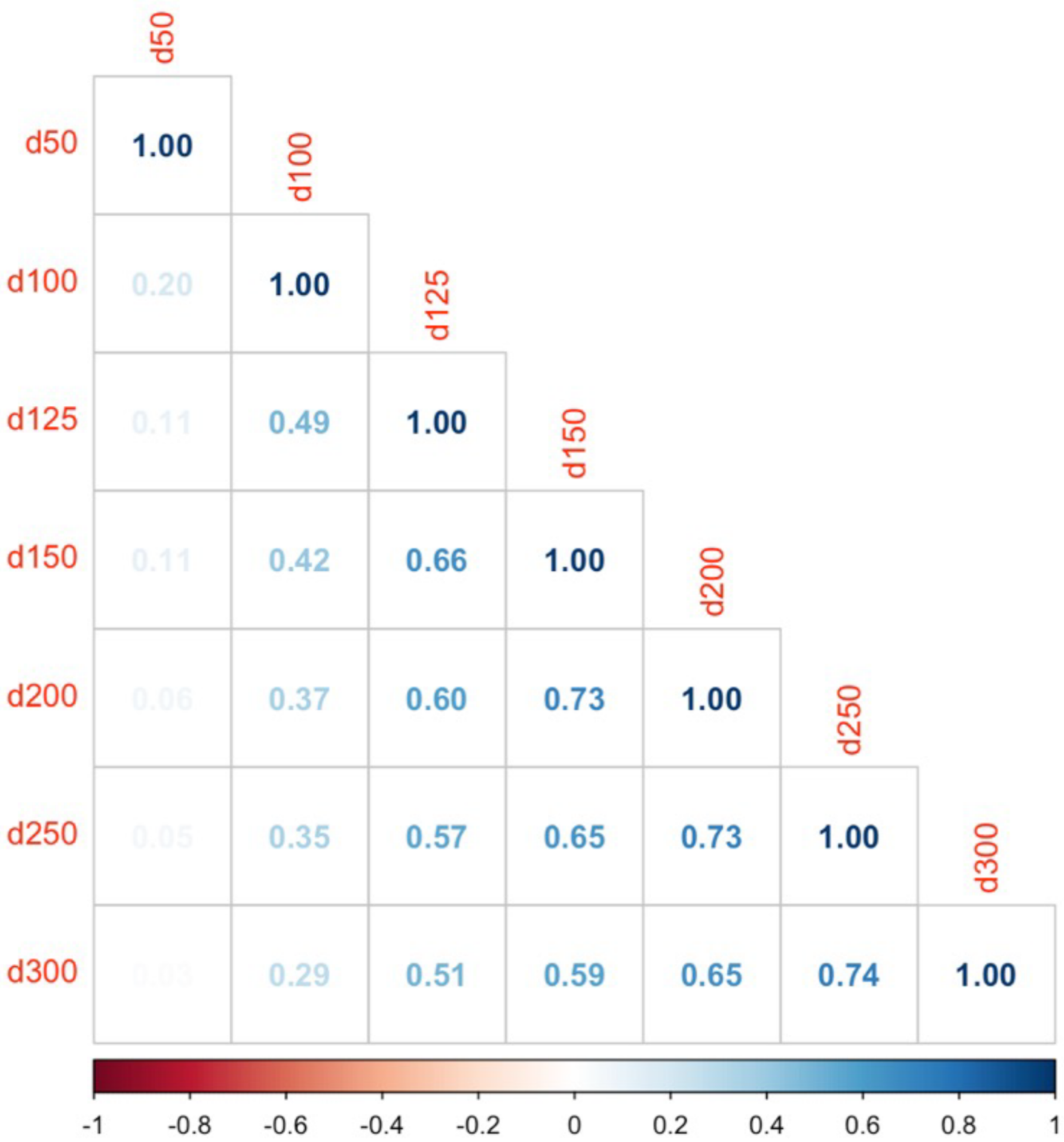

Supplementary Figure 1: Cophenetic correlation coefficients for the top 100, 125, 150, 200, 250, and 300 seed gene comparisons in Proteinarium between papillary and non-papillary patients for the TCGA Cell 2017 dataset (PMID: 28988769). Scores closer to 1 indicate greater similarity between the dendrograms.

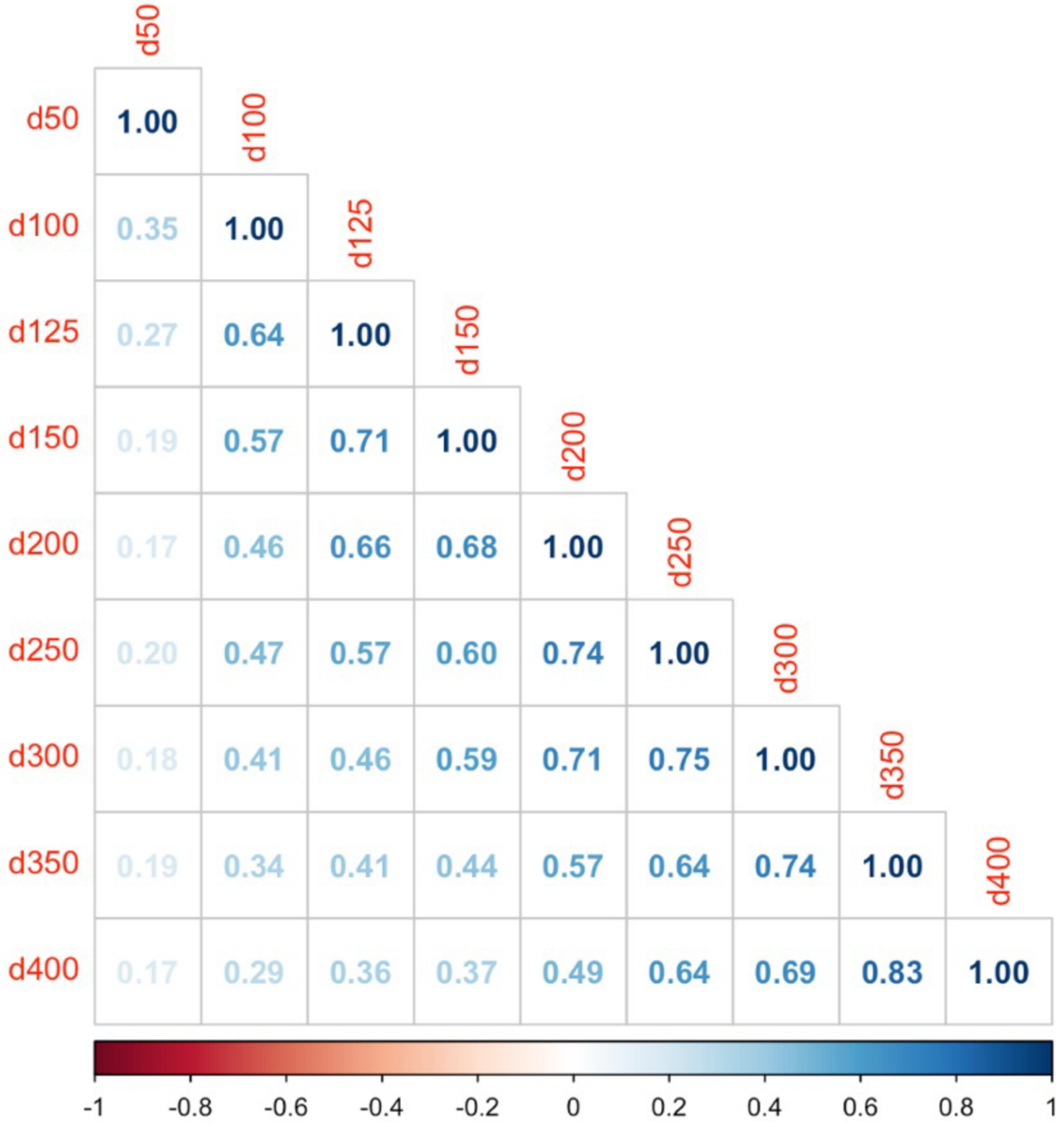

Supplementary Figure 2: Cophenetic correlation coefficients for the top 50, 100, 125, 150, 200, 250, 300, 350, and 400 seed gene comparisons in Proteinarium between papillary and non-papillary patients for the TCGA Nature 2014 dataset (PMID: 24476821). Scores closer to 1 indicate greater similarity between the dendrograms.

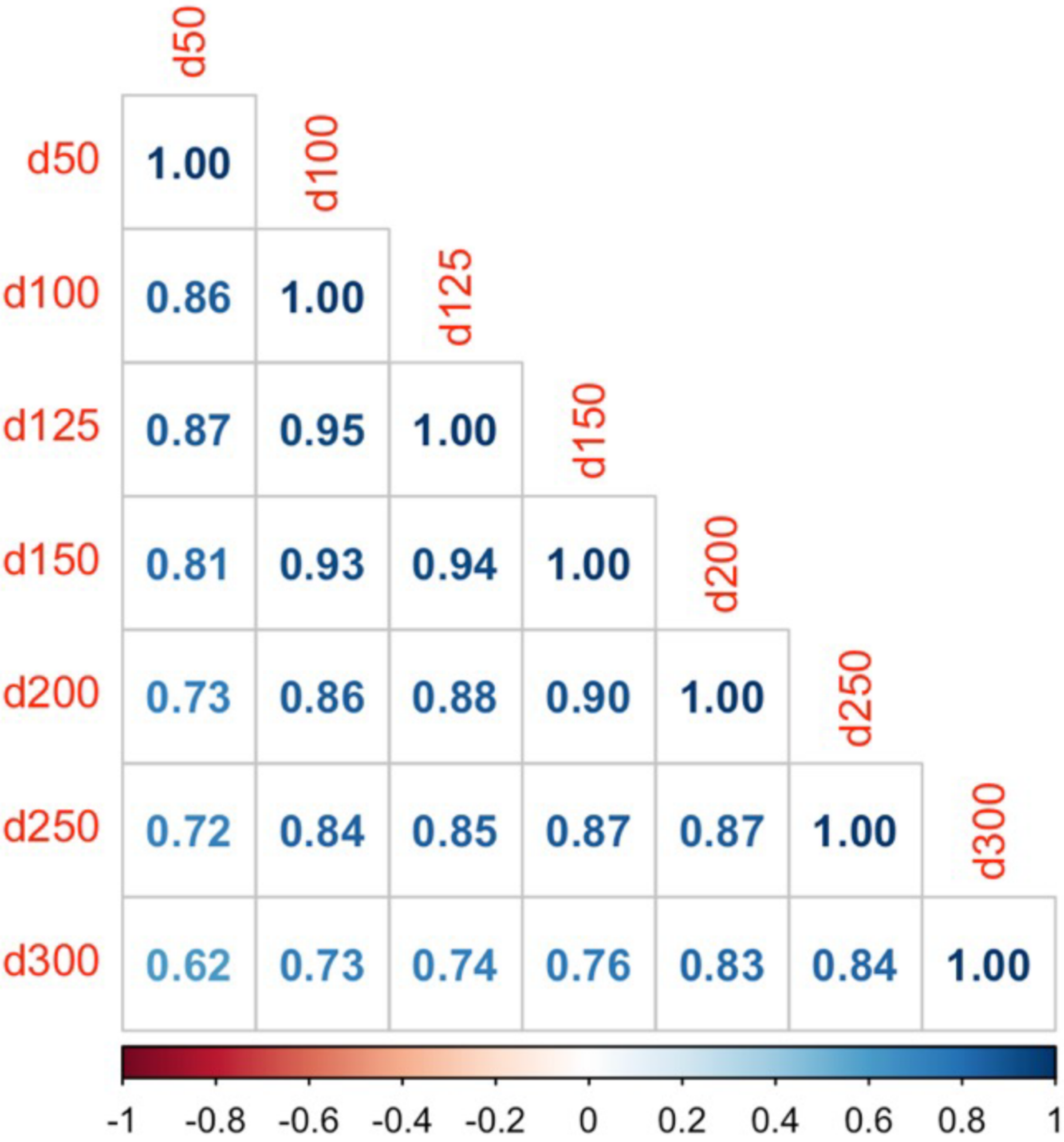

Supplementary Figure 3: Cophenetic correlation coefficients for the most downregulated 50, 100, 125, 150, 200, 250, and 300 seed gene comparisons in Proteinarium between papillary and non-papillary patients for the TCGA Cell 2017 dataset (PMID: 28988769). Scores closer to 1 indicate greater similarity between the dendrograms.

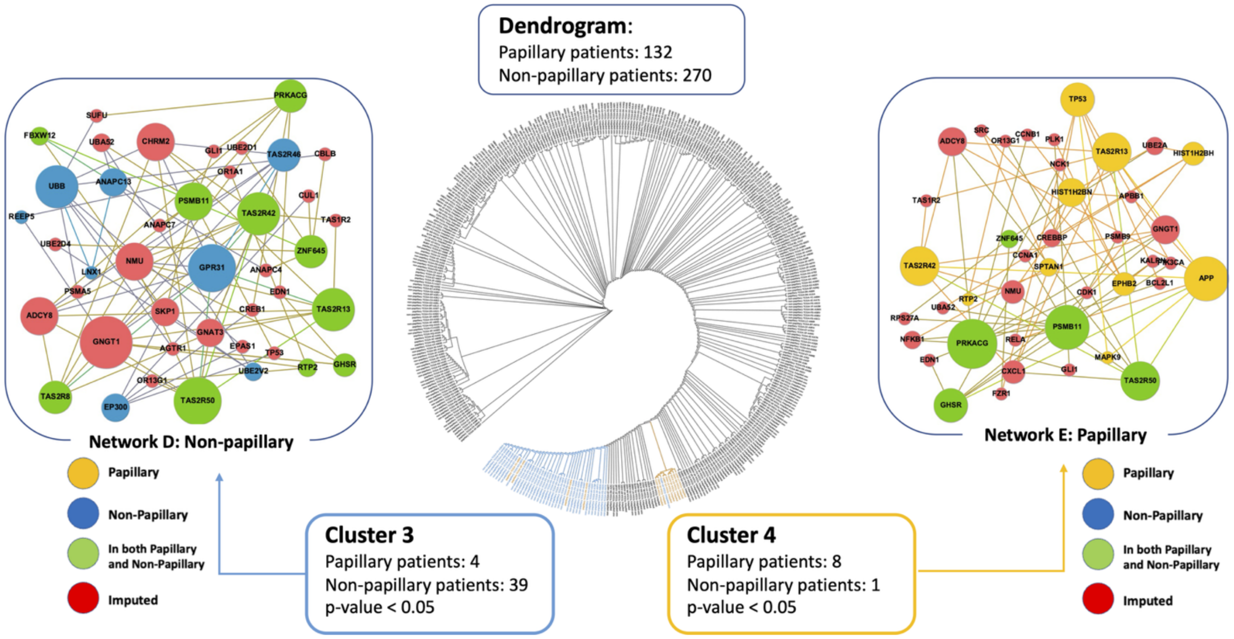

Supplementary Figure 4: Dendrogram and consensus PPI networks of the papillary and non-papillary urothelial carcinoma patients using the 125 most downregulated genes.

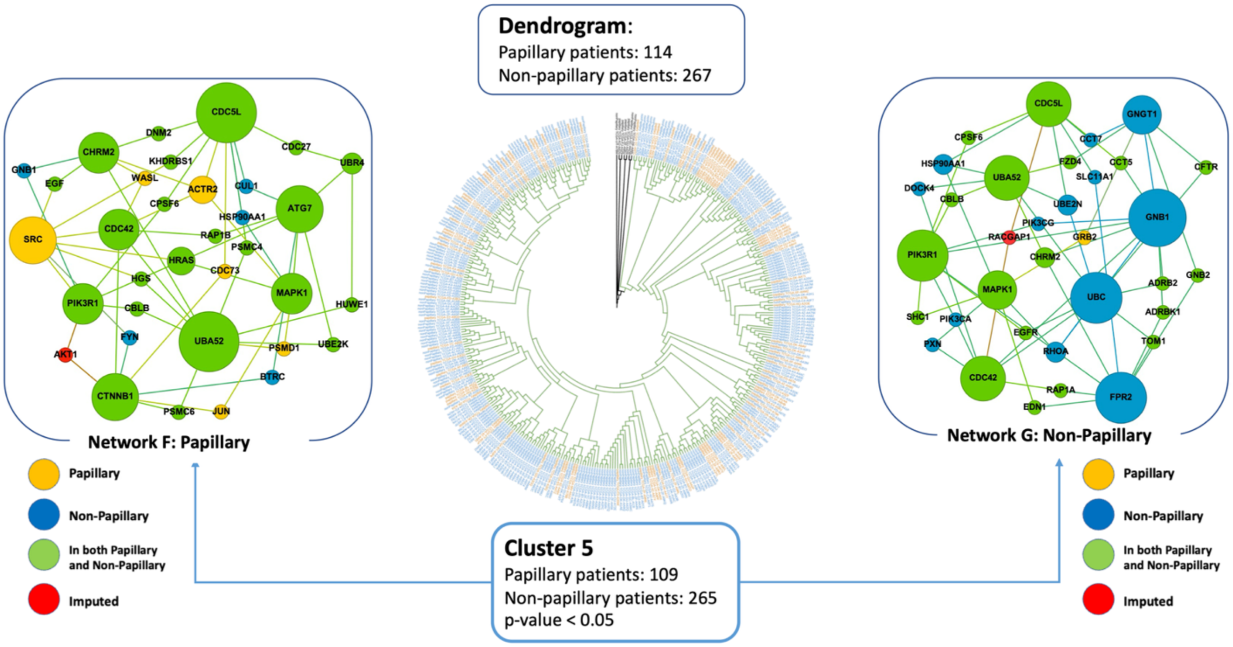

Supplementary Figure 5: Dendrogram and consensus PPI networks of only patients with high-grade papillary (n = 114) and high-grade non-papillary urothelial carcinoma (n = 267).
