# Supplementary material for: Protein-Protein Interactions in Papillary and Nonpapillary Urothelial Carcinoma Architectures: Comparative Study
Source: JMIR Bioinform Biotechnol. 2025 Nov 27;6:e76736. doi: 10.2196/76736 (PMC12661593; doi:10.2196/76736)
Supplement: Multimedia Appendix 2 [file bioinform-v6-e76736-s002.zip › Files for Reproducibility/Proteinarium Settings.rtf]

Proteinarium Settings:Max Number of Vertices to Render: 50Meta Cluster Threshold: 0.8Number of Bootstrapping Rounds: 0Max Path Length: 2Repulsion Constant: 1.2
